# Isolation and Characterization of Seneca Valley Virus From Pig Transboundary Spread to the Mink Infection

**DOI:** 10.1155/tbed/4428550

**Published:** 2025-06-16

**Authors:** Ziliang Qin, Xinmiao He, Chao Chen, Shaojun Chen, Zida Nai, Yao Wang, Wentao Wang, Gang Li, Fang Wang, Ming Tian, Haijuan He, Heshu Chen, Di Liu, Xinpeng Jiang

**Affiliations:** ^1^College of Animal Science and Technology, Northeast Agricultural University, Harbin, Heilongjiang, China; ^2^Institute of Animal Husbandry, Heilongjiang Academy of Agricultural Sciences, Harbin, China; ^3^School of Life Sciences, Peking University, Beijing, China

**Keywords:** cross-host transmission, mink, Seneca Valley virus

## Abstract

Seneca Valley virus (SVV) infection has recently disseminated across pig farms in Canada, America, and China. The SVV has been identified in humans, rodents, and houseflies. Although cross-species transmission events may lead to limited subsequent transmission, sustained outbreaks have been observed in new mammalian hosts. Thus, in our study, we utilized molecular characteristics, pathological examination, and the immune response to ascertain whether mink could serve as a novel mammalian host for SVV. Here, our study utilized a porcine strain of SVV to infect minks orally, resulting in pathological changes in the intestines. In addition, SVV could stimulate a specific neutralizing antibody response. The neutralizing antibody against SVV has also been found in mink through an epidemiological investigation in Heilongjiang Province. This study highlights the role of SVV infection in minks as an impetus for viral evolution, which poses potential threats to livestock, public health, and economic prosperity.

## 1. Introduction

Picornaviruses commonly infect a wide range of animals and humans. They cause broad clinical symptoms, such as myocarditis, meningitis, encephalitis, diarrhea, and paralysis. Recently, research has focused on the dependency between host transmission and picornavirus infection in the intestines. Many studies have shown that picornaviruses break host barriers that are closely related to the genetic similarity between species. The different viral genera can break their adaptation to new hosts, such as kobuviruses, foot-and-mouth disease virus, and enterovirus [1–3]. Seneca Valley virus (SVV) is a single-stranded positive-sense RNA that belongs to the genus *Senecavirus* within the family Picornaviridae, which is closely related to the genus *Cardiovirus*, and they are known for infecting vertebrate animals, including pigs, mice, and humans. Histopathology revealed that SVV caused pathological changes in epithelial cells and acute lameness myocarditis, such as vesicular lesions, interstitial pneumonia, and atrophy of intestinal villi with vacuolation of superficial epithelial cells [4, 5]. The virus has been demonstrated to be able to spread to other internal organs without any other clinical manifestations.

The concept of “sentinel species” in cross-species transmission is important in public health sciences because sentinel species can provide the best animal models for further research on the Seneca virus(SV), offering integrated and relevant information on virus evolution through adaptive mutations and neofunctionalization [6, 7]. The mink (*Mustela vison*), a member of the weasel family, is a carnivorous mammal that has a high trophic status in the wild. Its diet consists of a wide variety of animals, including mice, frogs, snakes, birds, and small mammals. The mink has been found with many virus infections through research on cross-species transmission. Recent studies on the SARS-CoV-2 virus concluded that mink could be the host [8, 9].

Interestingly, mink, a neglected mammalian host, is infected by more subtypes of influenza A viruses, including both mammalian influenza A viruses and avian influenza A viruses [10, 11]. According to an epidemiological survey, mink may be an important sentinel species for virus surveillance and early warning. However, many major problems related to the dynamics of cross-species transmission in the models in relevant settings still need to be solved. More importantly, most studies have relied on prospective inference and reconstruction during infection without mechanistic research on immunity [12].

The SVV used in this study was from a farm and was used to infect mink and describe the associated clinical signs and pathological and virological findings. Sequence analysis of SVV revealed its role as a probable source of initial infection, indicating that SVV is transmitted between mink, mice, and pigs and is also hazardously excreted by mink in the environment for persistent infection in the wild. This study is the first to detect cross-species transmission with SVVs in minks.

## 2. Materials and Methods

### 2.1. Ethics Statement

This study abided by the animal welfare guidelines of the World Organization for Animal Health. All of the clinical animal samples used in this study were approved by the Committee on Ethics from the Animal Science and Technology College of Northeast Agricultural University for routine testing. The animal health code used was NEAU201918.

### 2.2. Cell Reagents and Virus Isolation

**Figure 1:**
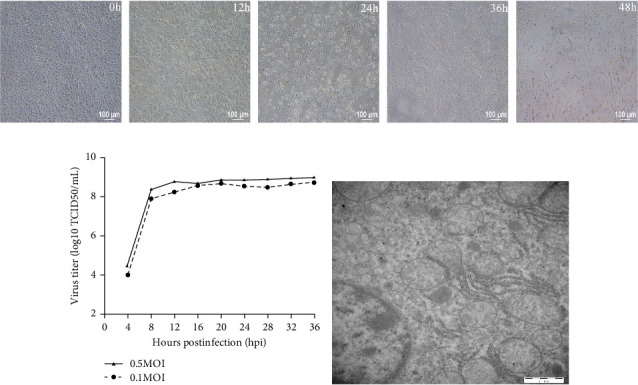
The process of BHK-21 cell CPEs induced by SVV-CH-09-2018 infection of cells (A). One-step growth curve of SVV-CH-09-2018 on BHK-21 cells with multiple infections (MOIs) of 0.1 and 0.5 (B). An electron microscope image of the SVV (C).

Baby hamster kidney 21 (BHK-21) cells cultured in Dulbecco's modified Eagle's medium (DMEM; Invitrogen, Carlsbad, CA, USA) were grown at 37°C in a humidified 5% CO_2_ incubator. The BHK21 cell line was utilized for the proliferation of SVV via tissue samples obtained from infected piglets on a farm in Heilongjiang Province, from which our laboratory isolated the SVV strain found in the vesicular sample collected from the infected piglets. The SVV viral RNA was extracted from the vesicular sample, which was subsequently converted into cDNA via the reverse transcriptase HiScriptQ RT SuperMix (+gDNA wiper) and synthetic cDNA primers (Vazyme, Nanjing, China). The cDNA was used as a follow-up template for PCR analysis of SVV-specific primers. The purified PCR products were subsequently sequenced (GenScript, Nanjing, China). The primers for SVV-1/2 were used to amplify the VP1 of SVV, and other vesicular disease viruses, such as FMDV (serotypes Asia 1, O, and A), VSV, SVDV, and VESV, were amplified via RT-PCR. The method of virus isolation was used with BHK-21 cells, as previously described. The infectious samples were harvested for 2 days until the cytopathic effect (CPE) was observed [13] (Figure 1A). The harvested CPE was cultured in BHK-21 cells for examination of SVV via RT-PCR, as described above. The isolated strain was named SVV-CH-09-2018. SVV-CH-09-2018 was added to BHK-21 cells at MOIs of 0.5 and 1 to generate one-step growth curves [14] (Figure 1B).

### 2.3. Cell Preparation for Transmission Electron Microscopy

The stained SVV-CH-09-2018 was observed via transmission electron microscopy (TEM). Viruses of SVV-CH-09-2018 were used to infect BHK-21 cells for 18 h, which were subsequently washed with precooled PBS twice and fixed with glutaraldehyde at 4°C. The scraped cells were centrifuged at 2000 × *g* for 10 min, and the supernatant was discarded. The sample was used for postfixation in OsO_4_, which was embedded in epoxy resin and polymerized at 80°C for at least 3 days. Finally, the cell samples were cut into 60–nm slices for staining with uranyl acetate [15]. The grids were observed via TEM (Hitachi HT7600 TEM, Japan).

### 2.4. Sequencing and Phylogenetic Analyses

The SVV-CH-09-2018 whole genome was divided into seven overlapping fragments. Seven fragments were PCR amplified via Prime STARHS (TaKaRa, Dalian, China). The primers used are shown in Table 1. The PCR product was purified, cloned, and inserted into the pEASY-Blunt Simple clone vector (transgene), and the cloned products were sequenced by Sangon Biotech (Shanghai, China). The SeqMan II program in the DNASTAR software package (DNASTAR, Madison, WI, USA) was used to assemble seven overlapping fragment sequences into a complete genomic sequence via a 5'-full RACE core kit and 3'-full RACE core kit (TaKaRa, The specific primers of Dalian, China) were used to detect the 5'- and 3'-utr (Table 1), and the sequences targeted by the primers were designed on the basis of the existing SVV sequence with the PCR product sequence.

To construct a gene development tree, we first analyzed the SVV-CH-09-2018 gene sequence via BLAST. After obtaining and filtering similar sequences, we screened Seneca genes from several countries, including the United States, China, Canada, Vietnam, Colombia, and Brazil. We used MEGA7.0 and OMICSTUDIO evolutionary tree software for phylogenetic genome analysis. After aligning the selected blocks via Clustal, we used the neighbor–joining method. In the phylogenetic test, we used the bootstrap method, and the number of bootstrap replications was set to 1000. Evolutionary tree processing is continued in OMICStudio after the preliminary evolutionary tree is obtained.

### 2.5. Mink Challenge Assay

The SVV-CH-09-2018 strain was used for the challenge test. Minks that had been weaned for ~60 days (half male and half female) were selected for observation for 1 week to ensure that they were asymptomatic. SVV, FMDV, SVDV, VSV, and PRV were not detected by the corresponding ELISA antibody kit or RT-PCR or PCR methods. Two minks are divided into two groups. The first group was injected intraperitoneally with strain SVV-CH-09-2018 at 5 mL (1 × 109 TCID50/mL), and the second group was inoculated with DMEM as a negative control (NC). Both groups of mink were fed under the same conditions and in separate rooms, where strict biosafety protocols were followed to avoid crossover. The clinical symptoms of the patients were monitored daily for 28 days. At 0, 3, 7, 14, 21, or 28 days after the challenge (d.p.c.), mink serum was collected, and the anti-SVV neutralizing antibody titer was determined [16]. TaqMan real-time RT-PCR was used to detect the viral load [17, 18]. A standard curve was generated by plotting the threshold values against the serially diluted plasmid DNA encoding the SVV VP1 gene fragment. At 28 d.p.c., the mink was euthanized for pathological examination. The heart, spleen, liver, kidney, lung, inguinal lymph nodes, and other organs were removed for histopathological observation. As mentioned previously, TaqMan real-time RT-PCR was used to detect the viral titer in these organs.

### 2.6. Pathological and Immunofluorescence Examination

Intestinal tissues were collected for pathological and immunohistochemical examination within 10–15 min after the mink died. Intestinal tissue was impregnated with formalin for 4 h, and soaked in ethanol at different concentrations for 2 h. The samples were embedded in paraffin wax. The 6 µm paraffin sections were stained with hematoxylin–eosin (H&E) for histological examination via light microscopy (Olympus, Tokyo, Japan). The dewaxed sections were subjected to an immunofluorescence assay [19]. The sections were blocked with 0.3% bovine serum albumin (BSA) in PBS at room temperature (RT) for 3 h, and then washed with precooled PBS three times. After permeabilization with 0.4% Triton X-100 at RT, the sections were incubated for 45 min at RT with a specific anti-SVV antibody against VP1, which was produced in our laboratory. The secondary goat anti-mouse IgG H&L antibody (FITC) (Abcam) was incubated with the sections for 30 min at RT. Finally, the samples were washed and examined under a fluorescence microscope (Leica, Wetzlar, Germany).

### 2.7. Production of Specific SVV Antibodies

All the minks received the same feeding conditions. The patient's clinical symptoms were monitored daily for 28 days. At 0, 3, 7, 14, 21, or 28 d.p.c., the mink serum was collected to examine the quality of the specific antibodies via ELISA. The VP1 protein, which was produced in our lab, was obtained via prokaryotic expression. The secondary antibody horseradish peroxidase-conjugated (HRP) rabbit anti-Mink IgG/HRP (SolarBio, Beijing, China) was added, and the samples were then incubated for 1 h at RT and washed three times. The substrate o-phenylenediamine dihydrochloride (OPD) was used as the chromogen. The reaction was analyzed at 490 nm with an ELx800 microplate reader (BioTek, Winooski, VT, USA). The results of each group of plates were standardized via a panel of reference IgG negatives and positives. P/N ratios >2 were considered positive antibodies.

### 2.8. Assessment of Anti-SVV Antibodies in Mink Sera by ELISA

Heilongjiang Province is located in Northeast China and has a cold temperate zone and temperate continental monsoon climate, which is suitable for mink survival and breeding. Between June 2021 and May 2022, a total of 31 mixed samples from four mink farms located in Heilongjiang Province were collected. One farm was the foundation seed of a mink farm, and the minks were all introduced from Denmark in 2019 as SVV-negative serum samples. Blood was collected through jugular puncture from each mink in vacutainer tubes. In the field, the blood was allowed to clot before being transported to the laboratory in the district. At the district laboratory, the samples were centrifuged for 10 min at 3000 rpm to obtain the sera. Briefly, the VP2 gene was cloned and inserted into a *p*-Cold plasmid expressing the SVV structural protein, which was subsequently used to construct an ELISA method for detecting SVV-infecting antibodies. The NC sera showed no detectable VP2-specific antibodies via ELISA.

### 2.9. Statistics

All the data from the different groups were compared via one-way repeated-measures analysis of variance (ANOVA) and the least significant difference (LSD) test. Differences were considered statistically significant at *p*  < 0.05.

## 3. Results

### 3.1. Isolation and Purification of a Strain of SVV

The SVV, Teschovirus A, Sapelovirus A (SVA), Enterovirus G, FMD, VS, and SVD viruses were not detected in the vesicular outbreak clinical samples via the RT-PCR method; however, the expected 542-bp product size was detected in all vesicular fluid and swabs from pigs. With two sequential passages in BHK-21 cells, SVVs were successfully isolated from vesicular fluid samples. Two days postinoculation, the CPEs of infectious BHK-21 cells were found as cell lysates. No CPEs were evident in the NC cells (Figure 1A). SV was detected in cells via RT-PCR, and the other viruses were not detected in BHK-21 cells. The one-step growth curve of SVV-CH-09-2018 in BHK-21 cells was further examined. The multiplicities of infection (MOIs) of 0.1 and 0.5 were used to infect BHK-21 cells, and the infected cells were collected at 0, 4, 8, 12, 16, 20, 24, 28, 32, and 36 hpi. Titers were examined via the 50% tissue culture infective dose (TCID50) assay [13]. The virus replicates quickly at 4 hpi, and the highest titers peaked at 32 hpi. The maximum viral titer was the TCID50/mL (Figure 1B) [14]. After infection for 24 h, the virions in BHK-21 were round and had a diameter of ~30 nm according to electron microscopy (Figure 1A).

### 3.2. The Sequence of SVV-CH-09-2018 and Phylogenetic Analysis

**Figure 2:**
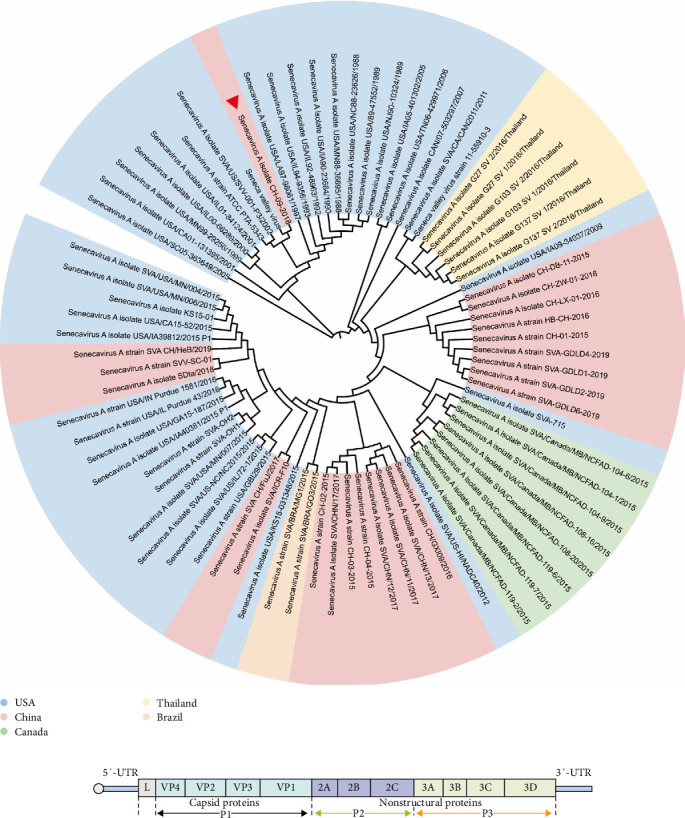
The evolutionary trees were drawn with MEGA 7.0 and Omicstudio, and the analysis data were obtained from the NCBI database with the whole genomes of the reference SVV strains. In the final evolutionary tree, the experimental virus strain SVV-CH is marked (▲), and the strains from different countries are also marked with different colors (A). Genome structure of the Seneca virus, showing the plane structure of virions. It is composed of a 5'-terminal noncoding region (5' UTR), a 3'-terminal noncoding region (3' UTR) encoding a polyprotein, and only one open reading frame (ORF) (B).

In our study, the evolutionary tree was drawn and analyzed by MEGA7.0 and OMICSTUDIO evolutionary tree software. We constructed this genome schema map on the basis of prototype SVV-CH-09-2018, which was isolated by our laboratory, as shown in Figure 2. The structure of the genome includes leader proteins, 5' and 3' UTRs, P1 region proteins, P2 region proteins, and P3 region proteins [20] (Figure 2B). The P1 protein constitutes capsid proteins, and the P2p3 proteins constitute nonstructural proteins [21]. The evolutionary tree revealed that SVV-CH-09-2018 has the typical L-4 genome layout of picornavirus. A previous phylogenetic analysis revealed that SVV strains can also be divided into four branches. The SVV-CH-09-2018 strain belongs to clade III [22]. SVV-CH-09-2018 shares the highest homology of the two countries, such as the American Senecavirus A (SVA) strain HB-CH-2016 (GenBank, KX377924.1) (99.66%) and the China SVA strain CH-01-2015 (99.67%) (GenBank, KT321458.1).

The tree was mainly screened for Seneca genes from the United States, China, Canada, Vietnam, Colombia, and Brazil, which indicates that SV is not an endemic disease in a small region but is present worldwide [23]. To clearly indicate the relationships among various strains in different countries, we used different colors to mark different strains from different countries (Figure 2A). The tree shows the spread of global SVV genomes in three major evolutionary clusters: USA-China-like and Canada-like strain clusters [22]. To date, more than half of the SVVs in China have been affected by SVVs in the United States, and some of the SVVs in China are from the United States and Canada. To obtain a clearer picture of how the SV spread around the world, we compiled the chronology of landmark incidents about SVV. The chronology was combined with the evolutionary tree to facilitate analysis of the evolution and prevalence of the Seneca molecular epidemiology study of SVV [24].

As shown in Figure 2 and Table 2, the SVV strains isolated by our laboratory were separated by different geographic locations and dates, suggesting that geographic distribution and the infectious host may contribute to the codon usage pattern in the evolution of SVA. Geographic dependency in viral strains often results from localized evolutionary pressures, such as environmental conditions and host-species interactions, leading to mutations that can alter codon usage. The geographic distribution and the host are the two main mutational pressures associated with natural selection [40]. However, the location where the strain in this study originated needed to be clarified. The first appearance of SV in China in 2015 marked a critical turning point in its epidemiology, as the outbreak led to a notable increase in the pathogenicity of the virus [25]. This initial outbreak was distinguished by significant changes in morbidity and mortality rates, highlighting the virus's evolving nature and its growing impact on affected populations. The strain of this study has been used for some time and has continuously evolved in China. Further investigations, including identification of the pathogenesis and molecular epidemiology, are urgently needed to study the evolution and prevalence of SVV.

### 3.3. Mink Infection of the SVV

The challenge experiments were performed in mink for 5 days. There were no dead mink after viral infection throughout the experimental process. The pathological results did not indicate frequent gross changes, such as changes in the surface of the lungs, ulcerative lesions, or liver, and kidney lesions. However, liquid feces was found on the second day after SVV infection, which indicated diarrhea in the small intestine of the mink. All the tissues were examined via RT-PCR and RT-qPCR to test the nucleic acid content of SVV. The RT-PCR results revealed that the oral fluid of mink (2/3) was positive, and all the fecal swab samples were positive [18]. The RT-qPCR method was used for the detection of RNA from SVV. The RT-qPCR results corresponded to the RT-PCR results for the nucleic acid level of SVV. However, many more RNA copies were detected in the fecal swabs than in the oral fluid samples in the infectious groups. There was no positive sample in the control group according to the RT-PCR and RT-qPCR results (Table 3).

### 3.4. Pathology and Immunofluorescence of SVV Infection

**Figure 3:**
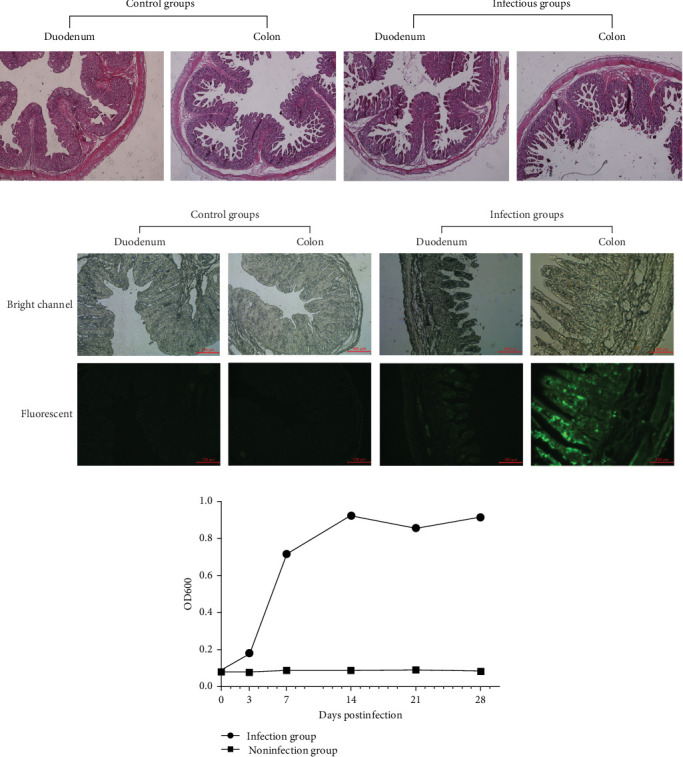
Pathology and immunofluorescence revealed pathological changes and the quality of the virus virion. Pathological changes in the duodenum and colon after SVV infection (A). Viral antigens were detected mainly in villous epithelial cells of the colon. The titer in the colon was much greater than that in the duodenum (B). In the mink challenge experiment, at 0, 7, 14, 21, and 28 days after the challenge, mink sera were collected to determine the level of anti-SVA neutralizing antibody (C).

Microscopic examination revealed obvious differences among the five groups of minks with respect to their small intestine samples taken from different intestinal segments, such as the duodenum and colon. The principal histopathological results are graphically summarized in Figure 3A. The intestinal sections revealed that the lesions were atrophied and ruptured in both the duodenum and the colon, followed by the fusion of villi in the duodenum and colon, which are also associated with inflammation in different intestinal segments [41]. The duodenum of the infected group was much more severe than that of the control group. In addition, there was necrosis and vacuolization of epithelial cells in minks, with clinical manifestations of diarrhea. The sections of both the duodenum and the colon in the control group did not exhibit any pathological changes [42].

The distribution and quantity of SVV virions in the small intestine of mink are shown in Figure 3B. The antigens of the SVV virion were identified via multiple antibodies against VP1 produced by our laboratory. The viral antigens were detected mainly in villous epithelial cells in both the duodenum and colon. We did not find viral antigens in the duodenum or colon of the control group. However, the viral antigens were detected in the duodenum and colon in the infected groups, and different intestinal segments contained different virus titers. The colon had much higher titers than did the duodenum with SVV infection, which showed that the epithelial cells of the colon were much more sensitive than those of the duodenum with SVV infection.

### 3.5. Quality of SVV-Specific Antibodies

All mink were fed under the same conditions. The patient's clinical symptoms were monitored daily for 28 days. At 0, 7, 14, 21, or 28 d.p.c., mink serum was collected to measure the anti-SVV neutralizing antibody titer in the two groups (Figure 3C). The SVV antibodies in the infected group were detected and measured at 28 days. There was no difference in the antibody-negative group. In the infection group, the number of antibodies gradually increased from 7 to 21 days. The antibody titer peaked at 21 days. The titer did not significantly decrease in the infectious group.

### 3.6. Serological Results

**Figure 4:**
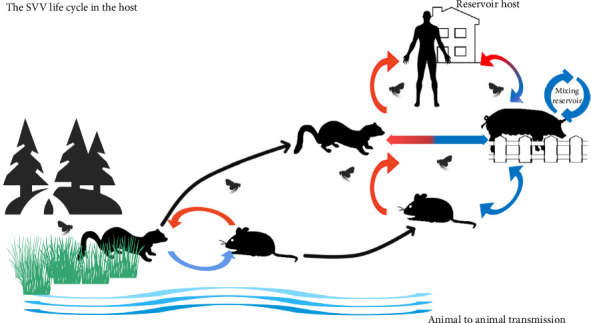
The schematic diagram of the adaptation and transmission of the SVV.

The sensitivity and specificity of the detection of VP2 from SVV were compared via ELISA in 32 clinical serum samples from four mink farms, one of which was a negative mink farm (Table 4). Overall, 48.2% (14 out of 29) of the tested sera were reactive to the SVV recombinant VP2 antigen. The proportions of the three mink farms were 0%, 60%, and 100%, respectively. One could attribute the occurrence of the SVV-reactive humoral response to similar VP2 proteins. There were significant differences in the levels of antibodies detected by ELISA in the serum samples collected from different farmed minks. This observation not only supports the existence of preexisting cross-immunity in the mink farm (Figure 4). However, another study revealed that the SVV could transboundary from pigs to minks. In our study, the sera used were from healthy subjects with no signs of infection, and 14 out of the 29 SVV-reactive sera were positive for anti-SVV IgG. The main reason was that farmed mink are also highly susceptible to infection by different viruses; often, the proportion of infected mink that have clinical disease is low.

An adaptation from pig SVV to human SVV includes two circulations: food chain circulation and reservoir circulation. In the reservoir and food chain circulation, SVVs are transmitted, mutated, and adapted between mice and minks (as well as other semiaquatic mammals). Minks and mice can be infected through contact with epidemic water. In a free stall barn system, usually in some areas of developing countries, pigs will inevitably lead to land habitat circulation, including that of human beings. The blue pathway is transmitted via the fecal route, whereas the red pathway is transmitted via the oral route. In rural areas of South Asia, Southeast Asia, Southern China, and Eastern China; pigs, mink, and mice, particularly chicks, are often observed to be eaten by humans. Pigs and mink also have the opportunity to eat mouse feces, but mice seldom eat pig feces.

## 4. Discussion

SVA, also known as porcine idiopathic vesicular disease, is a vesicular disease in pigs. It plays a significant role in the clinical and economic aspects of farm animal health. Recently, SVV outbreaks have been reported in numerous large swine-producing countries. It is similar to other important vesicular viruses, such as vesicular stomatitis virus, swine vesicular disease, and foot-and-mouth disease [4]. SVA has attracted special attention, with a focus on understanding its pathogenesis, immunology, and epidemiology, revealing several characteristics, including its impact on epithelial and epidermal cells, immunosuppression, immune evasion, and cross-host transmission.

In 2015, the first outbreak of vesicular lesions in newborn piglets was observed on farms in Guangdong Province, China [43]; this disease is associated with high mortality and was diagnosed as SVA infection. To date, more than half of the provinces in China have been affected by SVV infection, and the strain isolated from the northernmost province was the first of its kind to be studied. SVA has been identified for over 30 years since the first report from the United States, with a significant turning point in 2015, when several outbreaks of SVA-vesicular disease (SVA-VD) and epidemic transient neonatal losses (ETNLs) occurred [4, 44]. The first detected isolates were not associated with pathogenicity or clinical signs before 2010, whereas isolates identified after 2015 are considered “contemporary” owing to their association with vesicular lesions. Recently, phylogenetic studies have shown a significant divergence of 5.59% between our study strain and those isolated before 2010, indicating that SVV strains isolated before 2010 are considered “historical.”

SVV-001 was first detected in a PER. C6 fetal retinoblast cells were cultured in 2002, and it is believed that the virus was a contaminant from bovine serum or porcine trypsin in the cell culture [45]. The SSV sequences that have mutated over the past 10 years have undergone significant changes in their nucleotides and have also been found in different hosts and tumor cells. Mutational pressure from several animal hosts has accelerated the frequency of recombinant mutations in SVV [46], and cross-host transmission may have contributed to the rapid increase in the rate of these mutations [47].

In terms of pathogenesis, different types of SVVs, even those with sequences similar to those of strains from the USA, still show varying pathogenicity in pigs [43]. However, the replication efficiency of different strains remains consistently high. These characteristics suggest that SVV has the potential to infect various host animals. Notably, SVV has been detected and isolated from pigs, environmental samples, mouse feces, and mouse intestines. SVV RNA has also been detected in houseflies from farms without vesicular disease, which are located far from affected farms [34]. In 2012, a report indicated that SVV was associated with vesicular lesions, and a spontaneous outbreak occurred after pigs were purchased at the Indiana State Fair. SVV was first detected in China in 2015, with an outbreak occurring in 2016. The SVV strain in China represents the third major evolutionary cluster, alongside strains from the United States and Canada [44]. Additionally, all strains isolated from China could be grouped into clusters with United States and Canadian strains. As shown in Figure 2A, the isolated strain belongs primarily to the United States-like cluster.

In our study, mink was first used to study SVV infection, with RNA detected in oral fluid and fecal swabs via RT-PCR and RT-qPCR methods. Minks have become the fourth known infectious host, following humans, swine, and mice [48]. The RT-qPCR results indicated that the fecal swabs contained a greater quantity of SVV mRNA than did the oral fluid. Pathogenesis and clinical signs also revealed that the intestinal tract exhibited pathological changes and that there were no vesicular lesions in the mink following SVV infection. Histologically, finisher pigs presented multifocal pathological changes, such as infiltration of inflammatory cells, necrotic keratinocytes, and hemorrhage. Clinical evaluations in finisher pigs also revealed that the virus could be subclinically present, with some pigs not exhibiting clinical signs. The infiltration of inflammatory cells and necrotic keratinocytes was observed in experimentally infected pigs. Additionally, histopathological lesions, including interstitial pneumonia and ballooning degeneration of the urinary bladder and renal pelvis epithelium, are more severe in piglets than in finisher pigs [5]. These histopathological changes suggest that the SVV can invade epithelial and epidermal cells in mammals, such as pigs and mink. However, the exact mechanism by which SVV affects intestinal and oral epithelial cells in mink remains unclear.

The risk of SVA infection varies significantly between herds and farms, with many risk factors, such as the number of breeding females, the number of employes, and the timing of weaning, potentially contributing to the spread of SVV [49]. The analysis of serology in animals revealed that in 27 out of 71 porcine samples, neutralizing antibodies against SVV were detected; in 10 out of 30 bovine samples and five out of 35 wild mouse samples, the amount of neutralizing antibodies detected was no more than 100 human serum samples [27]. These data show that SVV can naturally replicate in farm animals and humans and that farm animals can be stimulated to produce neutralizing antibodies [50]. However, neutralizing antibodies are relatively rare in humans. Virus shedding was detected up to 28 days postinfection. However, studies have demonstrated that persistent viral shedding can be sustained up to 60 days after SVV infection [51]. The finisher pigs produced neutralizing antibodies at 5 dpi during experimental inoculation, and the maximum antibody concentration was between 7 and 14 dpi. However, the increase in neutralizing antibodies decreased during the first 2 weeks postinfection [24]. In a longitudinal study on SVA-infected farms, the antibody titers of piglets were greater during the first week of age but disappeared in the last four and 5 weeks. More importantly, 20%–40% of piglets with neutralizing antibodies presented viremia and viral shedding in feces and oral fluids, which was sustained between 4 and 5 weeks without clinical status [49]. In addition, studies on samples collected from swine and their environments at several sites revealed that high genetic diversity occurred in SVA over 12 months [52]. The special immune and infection status promoted mutation pressure, which was the main driver of the evolution of SVV rather than natural selection.

A sample from the swabs of internal and external surfaces on the farm was used for clinical evaluation, and the nucleic acid of SVV was detected, indicating that SVV is an environmental risker. The detection of SVAs in mice and houseflies indicated that SVVs may play a role in the epidemiology of SVV, increasing the risk of SVV infecting wild animals as natural hosts [34], and that mice may act as natural reservoirs and potential vectors. On the other hand, the minks were at the top of the food chain in the mice, which could increase the likelihood of SVV infection and the evolution of viruses. Previously, mutational pressure was considered the major factor influencing variation compared with natural selection. Most of these studies focused on the geographic distribution contributing to the codon usage pattern of SVA, and mutational pressure played a more important role in SVA evolution than natural selection [47]. However, no studies have focused on cross-species transmission, such as the complex links among the physiological differences of hosts, disease progression, and viral release. The ability of mink to infect SVV provides a new factor in mutational pressure, which could increase the understanding of the role of SVV in cross-species transmission and the viral life cycle within the environment, and humans could block the host in the spread of SVV. All of these studies provide scientific evidence for preventive measures against SVV.

SVV can evade the immune system not only in humans but also in animals. Surface antigen antibodies showed that SVV could stimulate the mink immune system, with antibody titers increasing after SVV infection. During the clinical evaluation period, there was no significant difference in IgG antibody dynamics between clinically affected and nonaffected animals. SVV was originally identified as a potent oncolytic virus against tumors in medicine, with features including the ability to target and penetrate solid tumors via intravenous administration, the inability to cause insertional mutagenesis, and self-replicating RNA viruses with selective tropism for cancer cells [53]. A strong cellular immune response is induced by SVV infection, which promotes the response of IFN-*γ*-specific T cells as early as 3–7 dpi [54]. Therefore, T-cell responses could not completely clear SVV at 14 dpi. However, there was no change in the evolution of SVV from the same infecting farm over 1 year of research, which indicated that the evolution was not from one pig host. Instead, multiple hosts contribute to the mutational pressure driving SVV evolution and cross-species transmission.

## Figures and Tables

**Table 1:** Primers for amplification and identification of the whole genome.

Primers	Sequences (5'–3')	Positions	Size (bp)
F (1-623)	TTTGAAATGGGGGGCTGGG	1–623	623
R (1-623)	CTATCAGGCAGTATCCAAAGCACGC	1–623	—
(498-1352)M13+	CGACCCAGGACTTCTTTTTGAAT	498–1352	855
(498-1352)M13−	GAGAAGGTTTTTACAGCT	498–1352	—
(1336-1662)M13+	GCTGTAAAAACCTTCTC	1336–1662	327
(1336-1662)M13−	ATAGTATGTGCCAAGAG	1336–1662	—
(1612-2748)M13+	GATTACCGGACCGGGAAAAACAT	1612–2748	1137
(1612-2748)M13−	ACCAGAGAAATCGGTGTCAGT	1612–2748	—
(2996-4041)M13+	CTTCACTGGACTTCAATTTTTATA	2996–4041	1046
(2996-4041)M13−	CTCCAACTGGTACTGGAGGACAG	2996–4041	—
(4004-4995)M13+	AAGAGAAAGCCAGCCCTGTCCTCC	4004–4995	991
(4004-4995)M13-	ACCTAGCTTGGCAAGAATAGCCAAACG	4004–4995	—
(4929-5949)M13+	GGCGCTTGTCGACCTCACTCCAGA	4929–5949	1021
(4929-5949)M13-	ATCAAATTTTGACAACACAGCA	4929–5949	—
(5879-6908)M13+	AATTGAGAAAGACGACCGCACA	5879–6908	1030
(5879-6908)M13-	GTCATCTTATACCCCAACTT	5879–6908	—
(6881-7297)M13+	GCGCTGCCAAGTTGGGGTATAA	6881–7297	417
(6881-7297)M13+	CCTTTTCTGTTCCGACTGAGTT	6881–7297	—
SVV-F	AACCGGCTGTGTTTGCTAGAG	59–79	147
SVV-R	GAACTCGCAGACCACACCAA	205–186	—
SVV-P	6/FAM-TCGAGAAGCTGCAATCTG/MGB-NFQ	143–167	—

**Table 2:** Chronology of the SVV landmark incident.

Detection	Species	Reference	Country	Incident
2002	Human (PER.C6)	[25]	USA	This virus was first discovered as a serendipitous finding in 2002, while cultivating adenovirus-5-based vectors in the cell linePER.C6
2007	Swine	[26]	Canada	In 2007, ~80% of 187 pigs shipped from Canada to the United States developed blister disease, and Senecavirus RNA was detected in these biological samples
2007	Human (neoplasms with neuroendocrine properties)	[27]	USA	SVV-001 has potent cytolytic activity and high selectivity for tumor cell lines on neuroendocrine properties versus adult normal cells. Systemically administered SVV-001 has potential for the treatment of metastatic neuroendocrine cancers
2008	Seneca virus (SVV-001)	[21]	USA	Complete genome sequence analysis of Seneca Valley virus-001, a novel oncolytic picornavirus
2012	Swine	—	USA	The United States reported the Seneca outbreak in pigs symptomatic vesicular
2015	Seneca virus	[28]		In 2015, the International Committee on the Classification of Viruses (ICTV) renamed SVV “Senecavirus A” (SVA) after the genus it belongs to, the Senecavirus
2015	Swine	—		The year 2015 was a turning point in the epidemiology of infections, with the massive global outbreak of Seneca
2015	Human (solid tumors)	[29]	USA	The SVA as an anticancer treatment, NTX-010, in phase I trials in children with relapsed/refractory solid tumors by Neotropix
2015	Swine	[20]	Brazil	The Senecavirus infect outside of North America
2016	Swine	[30]	China	The first identifification and complete genome of Senecavirus A affecting Pig with idiopathic vesicular disease in China
2016	Swine	[31]	Colombia	Emergence and whole-genome sequence of Senecavirus A in Colombia
2016	Swine	[32, 33]	Thailand	The first detection of Senecavirus A (SVA) in pigs in Thailand
2016	Mice and houseflies	[34]	USA	Detection of the emerging Picornavirus Senecavirus A in mice and houseflies, which may play a role in SVA epidemiology
2017	Swine feed	[35]	Brazil	Seneca Valley virus RNA detection in pig feed and feed ingredients
2018	Swine	[36]	Vietnam	First detection and genome sequence of Senecavirus A in Vietnam
2018	Human	[37]	Japan	Structural basis for anthrax toxin receptor 1 recognition by Seneca Valley Virus
2018	Swine	[38]	Brazil	A new wave of Seneca Valley virus outbreaks in Brazil
2019	Swine	[39]	USA	Developed a recombinant SVA strain (rSVAm SacII) using reverse genetics and assessed its immunogenicity and protective effificacy in pigs
Our study	Mink	—	China	The mink infected the SVV isolating from the pig

**Table 3:** RT-PCR results and CT ranges of qRT-PCR-positive samples.

Groups	Sample identification	SVV RT-PCR			SVV qRT-PCR (C_T_ range)		
		Oral fluid	Serum	Fecal swab	Oral fluid (21.8–35.9)	Serum (15.3–35.7)	Fecal swab (22.5–35.2)
Infectious group	Sample 1	(+)	—	+	34.9	35.9	25.6
	Sample 2	—	—	+	36.7	36.9	28.9
	Sample 3	(+)	—	+	35.6	36.1	23.9

Control group	Sample 4	—	—	—	37.1	37.7	36.2
	Sample 5	—	—	—	36.2	36.6	35.9
	Sample 6	—	—	—	37.7	38.1	36.1

**Table 4:** Anti-SVV antibody levels in mink sera.

Region	Negative	Positive	*p*-Value
Negative farm	3(100%)	0 (0)	*p* < 0.01
Farm 1	11(100%)	0 (0)	NA
Farm 2	4 (40%)	6 (60%)	—
Farm 3	0 (0)	8 (100%)	—

## Data Availability

The data that support the findings of this study are available from the corresponding author upon reasonable request.
